# Sound innovations for biofabrication and tissue engineering

**DOI:** 10.1038/s41378-024-00759-5

**Published:** 2024-11-19

**Authors:** Mengxi Wu, Zhiteng Ma, Zhenhua Tian, Joseph T. Rich, Xin He, Jianping Xia, Ye He, Kaichun Yang, Shujie Yang, Kam W. Leong, Luke P. Lee, Tony Jun Huang

**Affiliations:** 1 School of Mechanical Engineering, Dalian University of Technology, Dalian, 116086 Liaoning China; 2 Thomas Lord Department of Mechanical Engineering and Materials Science, Duke University, Durham, NC 27708 USA; 3 Department of Mechanical Engineering, Virginia Polytechnic Institute and State University, Blacksburg, VA 24060 USA; 4 Department of Biomedical Engineering, Duke University, Durham, NC 27708 USA; 5 Renal Division and Division of Engineering in Medicine, Department of Medicine, Brigham and Women’s Hospital, Harvard Medical School, Boston, MA 02115 USA; 6 Department of Biomedical Engineering, Columbia University, New York, NY 10027 USA

**Keywords:** Engineering, Physics

## Abstract

Advanced biofabrication techniques can create tissue-like constructs that can be applied for reconstructive surgery or as in vitro three-dimensional (3D) models for disease modeling and drug screening. While various biofabrication techniques have recently been widely reviewed in the literature, acoustics-based technologies still need to be explored. The rapidly increasing number of publications in the past two decades exploring the application of acoustic technologies highlights the tremendous potential of these technologies. In this review, we contend that acoustics-based methods can address many limitations inherent in other biofabrication techniques due to their unique advantages: noncontact manipulation, biocompatibility, deep tissue penetrability, versatility, precision in-scaffold control, high-throughput capabilities, and the ability to assemble multilayered structures. We discuss the mechanisms by which acoustics directly dictate cell assembly across various biostructures and examine how the advent of novel acoustic technologies, along with their integration with traditional methods, offers innovative solutions for enhancing the functionality of organoids. Acoustic technologies are poised to address fundamental challenges in biofabrication and tissue engineering and show promise for advancing the field in the coming years.

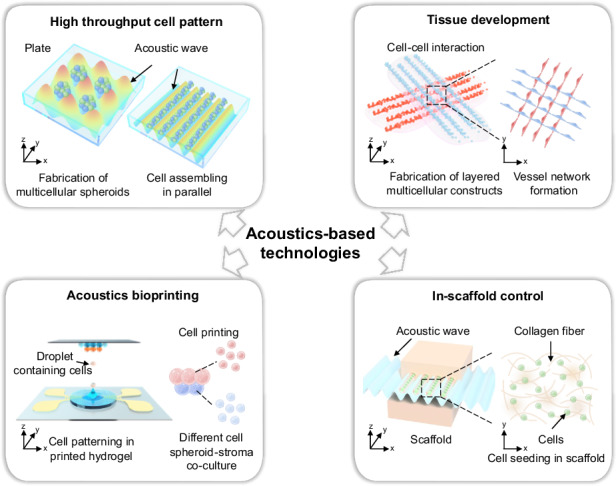

## Introduction

In Greek mythology, Prometheus shaped humans using mud, and Athena breathed life into clay figures. Similarly, in ancient Chinese mythology, Nüwa created human beings by squishing mud from a pond. The creation and organization of biological living things have been a common topic of mythology throughout history, and biofabrication, the modern-day Prometheus of bringing to life the ancient myths of creating life from molecules, biomaterials, and cells, is a heavily researched area of science today.

Biofabrication involves the generation of complex and biologically functional products from raw materials such as living cells, molecules, and other biomaterials^1^. The ultimate goal of biofabrication is to engineer artificial tissue constructs that not only replicate the structural complexity of native tissues but also emulate their functional capabilities. These constructs hold immense value for biomedical research, including the development of in vitro three-dimensional (3D) tissue models for drug testing and biological assays, which offer more accurate representations than traditional two-dimensional (2D) culture models^2–5^. Additionally, for in vivo applications, biofabrication promises to revolutionize regenerative medicine by creating tissue constructs that can repair or replace injured or diseased tissues and organs^6^. Despite significant progress, fully realizing biofabrication’s potential is an ongoing challenge, requiring continued research and development efforts to overcome existing limitations^7–10^.

State-of-the-art biofabrication methods can be divided into several categories based on the complexity of the fabricated constructs. The first category focuses on creating bioscaffolds from supramolecular materials, hydrogels, or other materials^11^. Cells are then seeded into the scaffolds for further culture, eventually leading to the formation of functional tissue constructs^12^. The second category involves generating multicellular spheroids or similar cell clusters, which can stand alone or as building blocks for more intricate tissue assemblies^13^. The third and most complex category involves sequentially fabricating and assembling scaffolds, cells, and cell clusters or directly forming tissues through methods such as 3D printing with cell-laden bioink^14,15^. As the complexity becomes closer to that of native tissues, greater demands are placed on the fabrication technology. Technology advancements in recent years have been encouraging, with various methods developed for all three aspects of biofabrication^10,16–18^. Among these methods, the acoustic-based method is one of the fastest-growing and can potentially address the challenges encountered in biofabrication and tissue engineering.

Acoustic technology has been widely used in the biomedical field for many years. One of the best-known examples is ultrasound imaging for diagnosis^19^. In addition, acoustic therapy has been approved for routine clinical treatment. It is used by physical therapists to accelerate the healing of bone fractures^20^ and stimulate the regeneration of cartilage^21^ and dentofacial tissues^22^. Other examples include focused ultrasound ablation^23^ and lithotripsy^24^, in which high-intensity pulsed ultrasound waves are applied to ablate solid tumors or break kidney stones while causing minimal harm to surrounding tissues^23,24^. These examples demonstrate that acoustic technology is a practical, biocompatible, and versatile tool for biomedical applications.

**Fig. 1:**
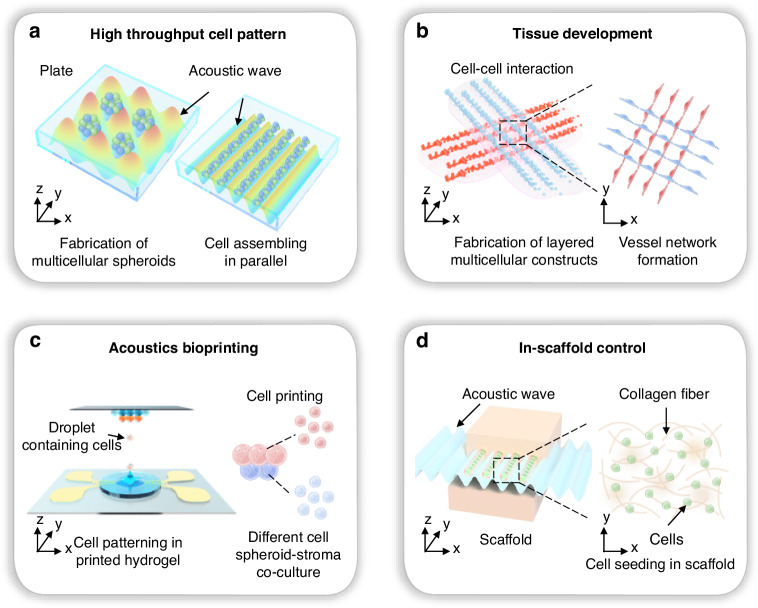
Unique features of acoustic technologies and their benefits for biofabrication and tissue engineering. **a** A high-throughput cell pattern can be achieved by acoustic wave periodicity. **b** Tissue development is initiated by layered assembly based on the periodicity of acoustic waves. **c** Acoustic bioprinting enables noncontact and gentle additive manufacturing due to the penetrability of acoustic waves in the fluid. **d** In-scaffold control of cells and scaffold structures becomes feasible because of the penetrability of acoustic waves in solid and gel-like materials

In addition to its biocompatible applications in biology, acoustic technology has two other unique characteristics that make it ideal for biofabrication and tissue engineering: periodicity and penetrability. These characteristics give acoustic technologies tremendous advantages in four aspects, as summarized in Fig. 1. First, the acoustic wave periodicity can naturally result in high-throughput parallel fabrication. This feature resolves the obstacles of high-throughput cell patterning and large-scale fabrication of spheroids and cell assemblies (Fig. 1a). Second, the acoustic wave periodicity enables the assembly of multilayered cell patterns. The three-dimensional-tuned acoustic fields make it possible to fabricate layered or other complex 3D constructs and provide an approach to study tissue development and organoid interactions (Fig. 1b). Third, acoustic waves can penetrate fluids to build an acoustic field in the native medium where cells live, thus increasing the convenience of noncontact manipulation. This feature suggests the possibility of a novel acoustics bioprinting method that does not rely on extrusion, thus offering less harm and better biocompatibility (Fig. 1c). Finally, acoustic waves can penetrate solid or gel-like materials, allowing the in-scaffold control of cells and supporting fiber structures. This ability extends the reach of cell manipulation from within the medium to most biomaterials used in biofabrication (Fig. 1d).

In the past decade, researchers have demonstrated the use of acoustic technologies for forming cell spheroids, spatial patterning of cells, fabricating scaffolds, engineering 3D collagen microvascular networks, and building complex 3D tissue constructs. For example, acoustic-based technology achieves higher throughput and shorter turnaround times than most other methods for forming spheroid-like multicellular clusters (Table 1). Furthermore, acoustic technologies offer excellent control of the size, biocompatibility, and accessibility of spheroids. These features have stimulated interest in the use of acoustic technologies for biofabrication and tissue engineering. Researchers are developing innovative engineering solutions to emerging problems in these fields. In this article, we first introduce the fundamental mechanisms of acoustic-based technologies. Then, we focus on the development of acoustic technologies over the past 20 years for various aspects of biofabrication and tissue engineering. Finally, we speculate on future directions for applying acoustic technologies in these fields.

**Table 1:** Comparison of different technologies for the formation of multicellular spheroids

	*Throughput*	*Spheroid formation time*	*Spheroid size*	*Cell-compatibility*	*Accessibility to spheroids*
Pellet culture^150^	Single spheroid in a centrifuge tube	2 h incubation on a shaker is needed	Only for large (millimeter scale) spheroids	Hypoxia causes necrosis in the spheroid core	Spheroids are harvested for further culture
Spinner flasks/rotating wall vessel^151^	Cells aggregate into spheres at 1 × 10^6^ cells ml^−1^ in 12 h.	12 h	Cannot control spheroid size	Cells may be damaged by shear forces	Easy to harvest spheroids, exchange medium, or add drugs
Hanging drop^152^	384 spheroids per run	Cells accumulate after 1 day	Controllable by adjusting the cell density	Good	Nontrivial to exchange medium or add drugs
Microfluidics/microwell^153^	200 spheroids per minutes	Spheroids formed in 1 day	Size-controlled	Good	Can exchange medium or add drugs; nontrivial to retrieve spheroids
Acoustic technology^29^	∼100 spheroids per hour, or more than 6000 spheroids per operation	Spheroids formed in several minutes to ~30 min	Controllable by adjusting the cell density	Good	Spheroids are harvested for further culture

## Acoustic mechanisms for arranging and assembling cells

When acoustic waves propagate through various media, such as cell culture media, hydrogels, tissues, and bones, they transport energy. Upon encountering cells suspended in diverse liquids, such as cell culture media, gelatin–methacryloyl (GelMA) bioink, and fibrin bioink, these waves exert acoustic radiation forces on the cells. The acoustic waves also induce acoustic streaming that further applies drag forces on the cells. Based on these forces, various acoustic technologies have been developed for enabling various cell manipulation functionalities, such as patterning cells^25–28^, concentrating cells^29–35^, separating and sorting cells^36–39^, and controlling cell‒cell distances^40,41^. Moreover, recent advances in acoustic-based cell manipulation have shown great potential in addressing the difficulties of current biofabrication methods, such as printing tissues with controlled anisotropic properties and constructing high-resolution patterns with single cells^29,42–46^. This section will briefly review the fundamentals of acoustic wave-induced forces for cell manipulation. In addition, this section will review the key acoustic-based cell manipulation mechanisms that can benefit biofabrication and tissue engineering. We expect this section to help biologists understand cell manipulation mechanisms and inspire engineers to develop next-generation acoustic technologies for biofabrication and tissue engineering.

### Acoustic radiation forces

When acoustic waves impinge on objects suspended in liquids, momentum is transferred, and primary acoustic radiation forces arise from the scattering of acoustic waves. Theoretical studies of the primary acoustic radiation force date back to King in 1934^47^, who found that the primary acoustic radiation force on a considerably small (i.e., the product of wavenumber and radius *kr* « 1) rigid sphere in an acoustic field is highly related to the monopole and dipole scattering. For a small compressible spherical object in an arbitrary acoustic field, Gor’kov subsequently developed a generalized potential-based theory to derive the primary acoustic radiation force F_*rad*_ expressed as^48^

1 $${{\bf{F}}}_{r}=-\nabla U$$

2 $$U=\frac{4\pi }{3}{r}^{3}[\frac{1}{2}{\alpha }_{1}{\beta }_{f}\langle {|p|}^{2}\rangle -\frac{3}{4}{\alpha }_{2}{\rho }_{f}\langle {|{\bf{v}}|}^{2}\rangle ]$$

3 $${\alpha }_{1}=1-\frac{{\beta }_{s}}{{\beta }_{f}}\,{\rm{and}}\,{\alpha }_{2}=\frac{2({\rho }_{s}/{\rho }_{f}-1)}{2{\rho }_{s}/{\rho }_{f}+1}$$

where $\nabla U$ is the gradient of the Gor’kov potential, *r* is the radius of the spherical object, *ρ* is the density, and *β* represents the compressibility, which can be calculated by the density *ρ* and pressure wave speed *c*. The subscripts “*s*“ and “*f*“ represent the sphere and the surrounding fluid, respectively. The terms <|p|^2^> and <|**v**|^2^> represent the mean squared values of the first-order pressure and velocity of the acoustic field, respectively.

The acoustic-based cell manipulation devices are typically operated in the Rayleigh region, where the cell sizes are substantially smaller than the wavelengths of the acoustic waves. The Gor’kov potential theory has shown great promise in determining the primary acoustic radiation forces on cells in this region^49,50^. Considering one of the typical cases, i.e., a cell in a standing acoustic field *p*(*z*, *t*) expressed by *p*_0_cos(*kz*)sin(*ωt*) (where *p*_0_ is the pressure amplitude, *k* is the wavenumber, and *ω* is the angular frequency), using the Gor’kov potential method, the primary acoustic radiation force can be derived as

4 $${F}_{z}=-{V}_{p}\,{E}_{{ac}}\,k{\Phi \; {\rm{sin}}}(2{kz})$$

5 $${E}_{{ac}}=\frac{{p}_{0}^{2}}{4{\rho }_{f}{c}_{f}^{2}}$$

6 $${\Phi }=\frac{5{\rho }_{s}-\,2{\rho }_{f}}{2{\rho }_{s}+\,{\rho }_{f}}-\frac{{\beta }_{s}}{{\beta }_{f}}$$

where *E*_ac_ is the average acoustic energy density and Ф is the acoustic contrast factor. This factor is critical in determining whether the target object is moved to a pressure node or an anti-node. When Ф < 0 (e.g., bubbles, some subgroups of lipoproteins), the acoustic radiation forces move objects to pressure antinodes. When Ф > 0 (e.g., fibroblasts in a cell culture medium), the acoustic radiation forces move objects to the pressure nodes of the standing acoustic field.

In addition to primary acoustic radiation forces, when cells are close to each other, secondary sources of radiation, or so-called Bjerknes forces, which arise from cell-induced scattering of acoustic waves, lead to nonnegligible effects on neighboring cells^51,52^. For example, these forces can enhance cell agglomeration by bringing together multiple cells in close contact. More details on the effects of secondary radiation on cells can be found in the work of Saeidi et al.^53^.

### Acoustic streaming-induced drag forces

In addition to acoustic radiation forces, cells in acoustic fields are subjected to drag forces induced by acoustic streaming. The acoustic energy dissipation in a viscous boundary layer can lead to so-called boundary-layer-driven streaming that highly depends on the acoustic device geometry. As acoustic waves propagate in a bulk liquid domain, the absorption of acoustic energy over a long distance can lead to a steady net flow along the wave propagation direction, known as Eckart streaming. The drag force induced by acoustic streaming can be expressed by

7 $${F}_{d}=6\pi \mu r{v}_{s}$$

where *μ* and **v**_*s*_ are the dynamic viscosity of the medium and the velocity of the cell relative to the medium, respectively. Although acoustic radiation forces accompany acoustic streaming-induced drag forces, most cell manipulation devices operate in regimes where the acoustic radiation force is dominant. Many devices that leverage acoustic streaming have also been developed to enable cell concentration, fluid pumping, and fluid mixing functionalities. Excellent reviews on detailed mathematical models and acoustic streaming mechanisms are provided by Wiklund et al.^54^ and Sadhal^55^, respectively.

## Acoustic-based high-throughput cell patterns

As mentioned previously, acoustic waves can control cells in a fluid medium through the primary and secondary acoustic radiation force or streaming-induced drag force. Given this nature, acoustic-based cell patterning has been widely applied in tissue engineering applications. Individual cells are the building blocks used for fabrication. Moreover, biomaterials such as hydrogels and collagen may be used as supporting glues. The patterned cells can eventually be assembled into spheroid-, band-, and network-like biostructures. Based on this classification, Table 2 summarizes recent progress regarding the assembly of cells using acoustic technologies. Various acoustic mechanisms have been thoroughly explored for each category, showing how acoustic-based technology can fit direct assembly and scaffold-based fabrication needs. Furthermore, the complexity of each acoustically assembled biostructure can be enhanced by utilizing methods such as periodically scaling up, duplicating the processing steps, or engineering the pattern of the acoustic waves.

**Table 2:** Summary of representative work on acoustic-based cell patterns

Biostructure	Cell types	Mechanism & Experimental setup	Medium/Scaffold	Refs.
Spheroid-like	Mammalian cells, HepG2	Half-wavelength acoustic resonator	Culture medium	^56–58^
	HepG2	Half-wavelength acoustic resonator	Alginate hydrogel	^59^
	Human red blood cells, HepG2	2D acoustic resonators formed by plane cylindrical, tubular or 2 orthogonal transducers	PBS, culture medium	^60^
	HepG2	2D standing surface acoustic waves	Culture medium	^29^
	Mouse embryonic carcinoma cells	1D standing surface acoustic waves in a single capillary	PBS	^61^
	Human MCF-7, A549, A2780, and murine embryonic carcinoma cell line P19	1D standing surface acoustic waves in microchannel arrays	Culture medium	^62^
	HeLa	1D standing surface acoustic waves in a capillary	10% PEGDA and 2% GelMA	^44^
	HepG2, A498, ACHN, and LUTC-2 cell lines	Microresonators	Culture medium	^63^
	BT-474 cells	Acoustic induced microstreaming in a 24-well plate	Culture medium	^35^
	C3A cells	3D transducer resonators	GelMA	^64^
	MCF7 cells	Acoustic streaming induced by a ring-shaped transducer	Culture medium	^65^
	MDA-MB-231 and MCF-7 cells	Acoustic streaming induced by sharp edges	Collagen	^67^
Band-like	Endothelial cells	Water tank with a transducer mounted at the bottom	Collagen	^69–71^
	C2C12, myoblasts	Multiwavelength resonator	GelMA, collagen	^43^
	Myoblasts	1D standing surface acoustic waves in a capillary	GelMA	^72^
	ASCs, MC3T3-E1 cells	Standing surface acoustic waves	Collagen	^73^
	HeLa	1D standing surface acoustic waves in a capillary	10% PEGDA and 2% GelMA	^44^
Network-like	C2C12 cells, Schwann cells	A heptagon standing acoustic field formed by 7 transducers	PBS	^74^
	Human adipose-derived stem cells or chondrocytes	Multiwavelength resonator	GelMA	^75^
	HUVECs and HvSMCs	Standing surface acoustic waves	GelMA	^76^
	Neonatal rat ventricular cardiomyocytes	Standing surface acoustic waves and a resonator in the vertical direction	GelMA	^45^
	HUVECs and hADSCs	Standing surface acoustic waves and a resonator in the vertical direction	HA-CA hydrogel	^46^

### Acoustic pattern for spheroid-like constructs

In the mid-2000s, several studies demonstrated the use of acoustic radiation to control cells and thus generate spherical cell aggregates in a simple and controllable manner^56,57^. A typical system setup can be found in a 2005 report by Bazou et al.^56^; this easy to build and inexpensive system consists of a piezoelectric transducer and a reflector, which form a half-wavelength acoustic resonator. A standing acoustic wave trap attributes cells suspended in cell culture medium to form aggregates and levitates the spherical cell aggregates in the acoustic resonator. The successful fabrication of mammalian cells and human HepG2 cell spheroids has been reported. Later, Bazou et al.^58^ examined the gene expression levels of mouse embryonic stem cells after exposure to acoustic standing waves and demonstrated that the standing acoustic wave field is minimally invasive and does not significantly affect gene expression. Hydrogel as a scaffold in acoustic-based cell assembly has many advantages, including the feasibility of fabricating more complicated structures and one-step formation. In 2008, Bazou et al.^59^ reported the use of a half-wavelength standing acoustic wave trap to form HepG2 cell spheroids in an alginate hydrogel. Kuznetsova et al.^60^ improved the setup by developing several types of resonators with periodic acoustic traps to increase the throughput of cell aggregate formation. Therefore, multiple spheroids could be formed in parallel. The cell aggregates created in one batch can reach 150–200 units using these enhanced setups. These pioneering studies demonstrate the potential of acoustic technologies in the biofabrication regime; however, bulky acoustic resonators make it challenging to control the size and repeatability of spheroids.

**Fig. 2:**
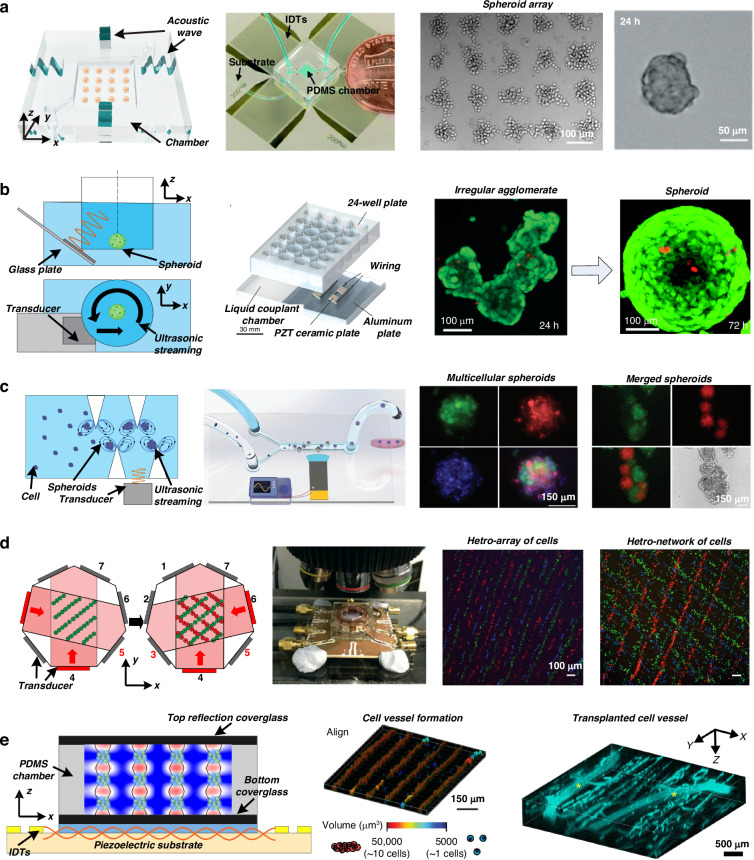
Acoustic technologies for cell patterning. **a** Formation of cell spheroids using 2D standing surface acoustic waves. The figures were reprinted and modified from ref. ^29^ with permission from the Royal Society of Chemistry. **b** Fabrication of cell spheroids in a 24-well plate using acoustic-induced microstreaming followed by culture for 72 h and confocal imaging of calcein/ethidium homodimer-stained cells. The figures were reprinted and modified from ref. ^35^ with permission from the Royal Society of Chemistry. **c** Sharp edges in the microfluidic channel induce microstreaming under the excitation of acoustic waves, thus aggregating cells to form spheroids. The figures were reprinted and modified from ref. ^67^ with permission from Wiley. **d** Dynamic patterning and formation of vessel-like and network structures in a heptagon acoustic tweezer. Cell band positions and directions are changed by applying a phase shift in acoustic waves or activating different transducers, respectively. The figures were reprinted and modified from ref. ^74^ with permission from the Royal Society of Chemistry. **e** Patterning and fabrication of a three-dimensional collateral distribution of vessels using a standing surface acoustic wave device. The fabricated cell-hydro 3D construct mimics vascular tissues. The figures were reprinted and modified from ref. ^46^ with permission from Springer Nature

Researchers have recently utilized microfabrication technologies to improve acoustic devices to achieve better resolution and higher throughput. One of the recent trends in the field is the use of surface acoustic wave-based technologies. Using microfabricated interdigital transducers (IDTs), surface acoustic wave devices have a higher frequency (typically 10–100 MHz) and more concise control of wavefronts and particle/cell positions. Chen et al.^29^ developed a rapid cell spheroid formation platform using two pairs of IDTs to generate a 2D acoustic pressure node pattern, as shown in Fig. 2a. The cell suspension was injected into the microfluidic chamber; the acoustic radiation force attracted the cells to migrate to the positions of the acoustic pressure nodes. The surface acoustic wave propagating on the substrate also induced acoustic streaming, which levitated the cells in the chamber. Therefore, cell spheroids were fabricated under the joint effects of acoustic radiation force and acoustic streaming. This platform continuously fabricated over 150 spheroids, which were transferred to Petri dishes every 30 min. With this high-throughput platform, the efficacy of antitumor drugs on tumor spheroids was tested. Wu et al.^61^ reduced the IDTs to one pair. A glass capillary was placed perpendicular to the acoustic pressure node lines formed by the IDTs. The cells in the capillary were pulled to aggregate and form spheroids. A reduction in the number of IDTs simplifies the electronics needed to power the device. However, it also decreases the throughput of spheroid formation. A study by Chen et al.^62^ used microfluidic channel arrays to replace a single glass capillary; thus, spheroids were generated in parallel channels. By scaling up the number of parallel channels, 12,000 pressure nodes were generated simultaneously within one microfluidic chip. The authors reported the ability to create more than 6000 tumor spheroids per operation. Downstream proliferation and drug testing experiments revealed the formation of a hypoxic core in the center of tumor spheroids and increased resistance to gemcitabine treatment compared to that in tumor cell monolayers.

Surface acoustic waves have also been employed for scaffold-based cell assembly. In 2016, Lata et al.^44^ used a standing surface acoustic wave device to align cells embedded in hydrogel fibers, mimicking physiological patterns found in tissues. By placing capillaries in perpendicular positions, they formed cell spheroids with precise spacing between them.

Other microfabrication techniques with acoustic forces can also enable the development of microresonators. Olofsson et al.^63^ etched silicon chips to form microreservoirs. Each reservoir serves as a microresonator when the chip is excited by an acoustic transducer. The microreservoirs also serve as isolated chambers for spheroids to grow individually. Therefore, the platform was used for the fabrication of multicellular tumor spheroids, downstream culture, and high-throughput screening. Miao et al.^64^ recently developed an acoustic device via three orthogonal piezoelectric transducers to generate three orthogonal standing bulk acoustic waves. They demonstrated that 3D-levitated acoustic nodes can be used to fabricate large-scale cell aggregates, with more than 13,000 produced per operation.

In addition to using acoustic radiation force as the primary driving force for cell assembly, Kurashina et al.^35^ demonstrated the use of acoustic streaming to form cell clusters in 24-well plates, as shown in Fig. 2b. By exciting the transducer immersed in the fluid, acoustic streaming is generated via attenuation of the sound propagating through the fluid. The streaming vortex flow-induced cell accumulation at the center bottom of the wells. Recently, Liu et al.^65^ developed a ring-shaped piezoelectric transducer that can be integrated with multiwell plates. They demonstrated the generation of circular standing flexural waves that induced acoustic streaming-driven cells to reach the center of each well to form cell spheroids. Mei et al.^66^ used focused IDT to excite acoustic streaming in a petri dish to aggregate cells in the culture medium. Rasouli et al.^67^ used oscillatory sharp edges inside microfluidic channels to generate acoustic streaming, as shown in Fig. 2c. This approach created closed acoustic vortices that trapped cells at the vortex center, forming cell aggregates. Furthermore, with the aid of collagen, the formation of multicellular spheroids and the merging of heterospheroids were demonstrated. Recently, Zheng et al.^68^ proposed the use of acoustically excited bubble arrays to form tumor spheroids. Bubbles were located in the microfluidic networks, and each bubble could induce microstreaming to trap and assemble the tumor cells. The authors demonstrated that the system could promote in situ drug response monitoring.

### Acoustic patterns for band-like constructs

Some pioneering studies regarding the use of acoustic technologies to fabricate vessel-like structures have been reported by Garvin et al.^69–71^. They assembled cells in collagen solution using 1D standing acoustic waves. When the cells were collected, collagen polymerization co-occurred, forming collagen gels with distinct vessel-like aggregated cell patterns. These results demonstrate a rapid, noninvasive approach to assemble cells and direct the formation of vascular networks in vitro.

In recent years, the technologies have been improved for better precision and more functions. Armstrong et al.^43^ used two transducers to pattern myoblasts in type I collagen. Myoblasts suspended inside a thin collagen layer formed vessel arrays with widths of approximately 60–80 µm after exposure to an ultrasonic standing wave. Then, these patterned materials were stimulated to undergo in situ myogenesis, and bundles of aligned myotubes were engineered. The authors demonstrated the formation of artificial muscle tissue, which exhibited anisotropic tensile strength, and the formation of muscle fibers containing aligned bundles of myotubes. Deshmukh et al.^72^ generated a standing acoustic wave field inside a glass capillary filled with a photopolymerizable hydrogel and cell suspension. The myoblasts within the hydrogel were patterned to form parallel lines to mimic the skeletal muscle structure. Villegas et al.^73^ developed a standing surface acoustic wave-based platform to pattern mesenchymal stem cells in collagen hydrogels into parallel lines. They demonstrated that the patterned cells exhibited enhanced metabolic activity.

### Acoustic fabrication of network-like constructs

Gesellchen et al.^74^ developed an electronically controlled acoustic tweezer device with a heptagon standing acoustic field formed by 7 transducers, as shown in Fig. 2d. Dynamic patterning of cell aggregates was created by switching the transducer excitation to change the orientations of the acoustic pressure nodes. Additionally, the trapping positions were shifted using a phase shift in the acoustic waves. The authors demonstrated the sequential handling of different cell types. The initial patterned cells were left to adhere to the substrate for ~30 min, followed by seeding another batch of cells at the newly positioned pressure nodes. Using this strategy, hetero-arrays and hetero-networks were formed with C2C12 cells stained with different fluorescent dyes. The authors fabricated Schwann cell band networks and cocultured them with rat dorsal root ganglia to guide neurite outgrowth. This study is useful as an in vitro model for peripheral nerve regeneration. Parth et al.^75^ developed a rotary fixture to install an acoustic transducer and reflector, enabling the orientation of the cell pattern to be altered by rotating the fixture. As a result, multiple patterns can be generated using a single transducer, significantly simplifying the complexity of the setup. Hu et al.^76^ reported the use of replaceable hydrogel frames to assist the fabrication of user-defined hierarchical or heterogeneous constructs through layer-by-layer assembly.

Naseer et al.^45^ proposed a surface acoustic wave-based mechanism to fabricate vessel-like structures. A sandwiched structure consisting of two cover glass slides and a layer of cell and photocrosslinkable GelMA hydrogel mixture in between was placed on a substrate with Fs. The IDTs generated standing surface acoustic waves, which aligned the positions of cells in the horizontal direction. Moreover, the two glass slides formed a resonator in the vertical direction, resulting in multilayered patterning of the cells. Later, Kang et al.^46^ improved the setup by replacing GelMA with HA-CA hydrogel. Figure 2e schematically depicts the mechanism involved. Simulations and experiments were conducted to refine the optimal design parameters by Naseer et al.^45^. Coaligned and multilayered human umbilical vein endothelial cell bands and human adipose stem cell bands were fabricated in the hydrogel matrix to form collateral microvessels^46^. The authors demonstrated the fabrication of vessel constructs, which enabled remarkable recovery of damaged tissue after implantation in an ischemia mouse model. The advances in these acoustic technologies hold promise for providing new methods for fabricating vascularized tissues for microphysiological research and regenerative therapies.

## Acoustic-based tissue development

### Acoustic fabrication of cartilage

Coakey et al.^77^ proposed a half-wavelength resonator to bring cell suspensions close together to form contacts at the position of acoustic pressure node planes. Initially, hexagonally packed cell monolayers were created, but then the hexagonal symmetry became unclear, and the cells formed dendritic aggregates with intercellular membrane spreading due to cell–cell interactions. Bazou et al.^78,79^ performed thorough studies using a half-wavelength resonator device to fabricate cell mats and investigate molecular adhesion development, gap junctional intercellular communication, and cytoskeletal organization.

**Fig. 3:**
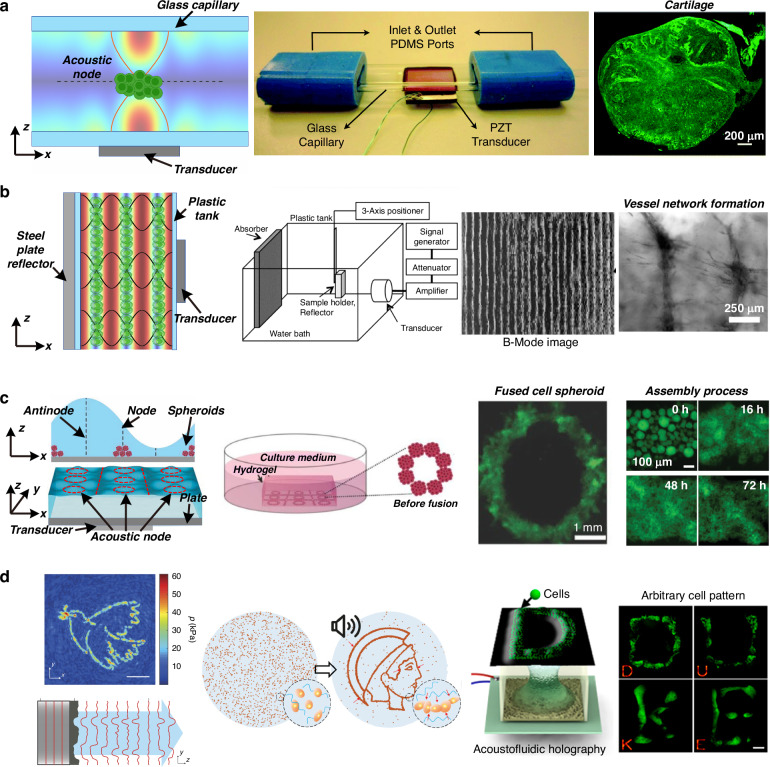
Acoustic technologies for tissue development. **a** Perfusion bioreactors and capillary-based acoustic resonators assemble human articular chondrocytes to form cartilage tissue after long-term culture. The figures were reprinted and modified from ref. ^80^ with permission from the Royal Society of Chemistry. **b** A multilayered membrane-like architecture is fabricated by a multiwavelength acoustic resonator and then cultured to form endothelial cell networks within collagen hydrogels. The figures were reprinted and modified from ref. ^82^ with permission from The Company of Biologists Ltd. **c** The use of Faraday wave patterns to fabricate heterogeneous multicellular membrane-like constructs. The figures were reprinted and modified from ref. ^86^ by John Wiley and Sons. **d** Hologram structures are used as acoustic masks to engineer the transmitted acoustic waves and form the designed pressure pattern. The figures were reprinted and modified from ref. ^92^ with permission from Springer Nature, ref. ^94^ with permission from John Wiley and Sons, and ref. ^95^ with permission from the American Chemical Society

In 2014, Li et al.^80^ demonstrated an acoustic technology for fabricating artificial cartilage tissue. As shown in Fig. 3a, a transducer is attached to a glass capillary to form a half-wavelength acoustic resonator, and the perfusion system introduces cells or chondrogenic medium. This system demonstrated the formation of a membrane-like structure with human articular chondrocytes and the development of neocartilage grafts after 21 days of culture. The fabricated neocartilage grafts were analogous to native hyaline cartilage and were implanted into the host cartilage to repair tissue defects. In 2018, the same group developed a second-generation design^81^. They investigated the mechanical stimulation induced by the acoustic field during graft culture and used these forces to modulate artificial cartilage with improved physical properties. These two studies demonstrated the ability of acoustic technology to fabricate and engineer implantable cartilage tissue. Multilayered membranes can also be manufactured by using multiwavelength acoustic resonators.

### Acoustic fabrication of vessels

In 2017, Comeau et al.^82^ applied a multiwavelength acoustic resonator to volumetrically pattern endothelial cells within collagen hydrogels, as shown in Fig. 3b. Multilayered cell constructs were fabricated throughout the hydrogel. Microvessel networks were formed after ten days of culture. The authors also demonstrated that the morphological features of microvessel constructs can be controlled by altering acoustic parameters such as frequency and amplitude. Mazzoccoli et al.^83^ used a cylindrical transducer to generate a radial standing acoustic wave field in which cells were assembled in a poly (ethylene glycol) diacrylate (PEGDA) solution. After UV exposure and PEGDA polymerization, multilayered cylindrical cell-hydrogel composites were fabricated. Armstrong et al.^84^ reported organizing living chondrocytes into high-resolution anisotropic arrays ~1–2 cells wide. This cytoarchitecture was maintained for five weeks in vitro, producing hyaline cartilage with cellular and extracellular matrix organization similar to that of the deep zone of native articular cartilage. Similarly, Comeau et al.^85^ applied this technology to in vivo tissue engineering and demonstrated that endothelial cells injected into the flanks of immunodeficient mice could be directly patterned in vivo using acoustics.

### Acoustic fabrication of organoids

Instead of acoustic resonators, Chen et al.^86^ utilized the Faraday wave pattern excited by a vibrating plate to fabricate organoids with various geometries, as shown in Fig. 3c. The spheroids were generated by culturing cells on a low-adhesion culture plate. Then, the spheroids were patterned and assembled in an acoustic standing wave field. This technique enables the assembly of many cell spheroids (>100) within a few seconds to form Faraday patterns, resulting in the formation of unique complex organoid structures. Later, Serpooshan et al.^87^ reported the use of this Faraday wave-based method to fabricate complex stem cell-derived cardiac tissue constructs. Patterned membrane-like structures were formed in the fibrin prepolymer and then maintained in culture media. The fabricated cardiac organoids exhibited significantly greater levels of contractile stress, greater beat frequency, and greater contraction–relaxation rates than did the constructs with random cell distributions.

Wang et al.^88^ used this method to fabricate functional biorobots composed of live cardiomyocytes. They demonstrated that the biorobot exhibited autonomous beating and could be integrated with synthetic skeletons to form a hybrid self-powered soft actuator. Recently, Gu et al.^89^ reported the assembly of stem cell-derived liver spheroids and endothelial cells into hexagonal cytoarchitectures. They found that hepatic lobule-like constructs can mimic the functions of the liver, such as albumin secretion and urea production. Marzio et al.^90^ used the Faraday wave pattern to fabricate ring-shaped microcapillary networks in fibrin hydrogels. A heterotypic tumor spheroid was surrounded by a vascular ring and treated with anticancer drugs, providing a model system for tailored tumor therapy. The same group also reported the assembly of anisotropic osteoinductive constructs and layered cellular structures^91^.

### Acoustic holography

Due to the periodic nature of acoustic waves, it is convenient to scale up the throughput by parallel processing and build layered or periodic constructs using acoustic technologies. However, it also presents a challenge to build arbitrary and nonperiodic constructs, which are preferable in some cases. For example, ellipsoidal constructs might more effectively mimic complex tissue geometry in vivo than spherical constructs.

A recent innovation—acoustic holography—has demonstrated the potential to solve the problem of fabricating biostructures with arbitrary cellular arrangements, as shown in Fig. 3d. In 2016, Melde et al.^92^ used a hologram plate to engineer the transmitted acoustic waves. They converted a specific pattern to a phase distribution using the iterative angular spectrum approach. Then, they encoded the phase information to the topography of the hologram structure. The plane wave generated by the transducer was diffracted after the hologram structure. The designed pattern was reconstructed as the acoustic pressure distribution at a certain plane. In 2018, Melde et al.^93^ demonstrated the use of this technique to assemble silicone particles into arbitrary 2D shapes. Ma et al.^94^ demonstrated the pattern and assembly of cell aggregates. The hologram structure modulates the acoustic waves generated by a transducer. The attenuation of acoustic waves induces localized microstreaming, and the resultant convection flow pulls the cells to form the designed pattern. Gu et al.^95^ used a binary phase acoustic hologram structure to demonstrate the assembly of cells with resolution at the single-cell level. They used a digital hologram structure, which led to a much finer resolution. A Petri dish was placed on top of the tank, and the acoustic wave engineered by the digital hologram structure could trap the cells in the Petri dish at positions that formed the letters “D”, “U”, “K”, and “E”.

Most recently, Ghanem et al.^96^ designed a phase hologram based on diffraction theory and an iterative angular spectrum approach to shape the acoustic field in 3D space. They achieved an acoustic pressure pattern similar to that of standing waves. Melde et al.^97^ combined multiple acoustic holographic fields and achieved the formation of whole 3D objects in a single attempt using acoustic forces. Recently, Xu et al.^98^ reported a medium-sound-speed modulation method to change the pattern of acoustic holography. They encoded multiple images onto a holographic phase plate. Adjusting the sound speed using an intervening fluid medium produced varied acoustic field distributions. These studies show emerging potential in tissue engineering for the fabrication of biostructures that mimic complex tissue geometries. Among the existing biofabrication and tissue engineering methods, 3D bioprinting methods can be used to reconstruct tissues and organs with complex geometries and structures^99,100^. However, existing 3D bioprinting methods must improve the resolution, cell density, and cell viability after printing^99^. Furthermore, compared with 3D printing or other methods in which each block is individually added, acoustic holographic technology can be used to fabricate an entire construct simultaneously, significantly reducing the turnaround time.

Jiménez-Gambín et al.^101^ demonstrated the formation of acoustic fields with complex spatial distributions inside the skull. By programming the hologram design, the distribution of the acoustic pressure matched the 3D structures of the central nervous system. This work illustrates that acoustic holography can be used to construct 3D objects in vitro and can be extended to in vivo applications due to the penetrability of acoustic waves.

## Acoustic-based 3D bioprinting

Tissues or organs usually have complex structures and geometries. Although it is possible to induce the development and growth of simple constructs (e.g., spheroids or layered composites) by adding initiators and thus eventually obtaining complex and transplantable tissues, the processes still need to be completed on time. Preconstruction of organ- or tissue-like constructs from cells and scaffolds will shorten the culture time, increase the throughput, and increase the consistency of the fabrication process. Therefore, it is important to have a method in which complex constructs can be built. The innovations in acoustics-based bioprinting offer solutions to resolve this issue.

Xu et al.^102^ presented a method to assemble complex cell constructs after polymerization. The cells were suspended in the hydrogel solution, and then, the hydrogel was polymerized into microblocks by lithography. Self-assembly of these cell-encapsulated hydrogel blocks occurred under the excitation of acoustic waves due to the secondary acoustic radiation force. Due to the lock-and-key design of the hydrogel blocks, complex constructs could be fabricated. Furthermore, a multilayer construct made of hydrogel blocks was fabricated with cells encapsulated using a layer-by-layer building strategy.

**Fig. 4:**
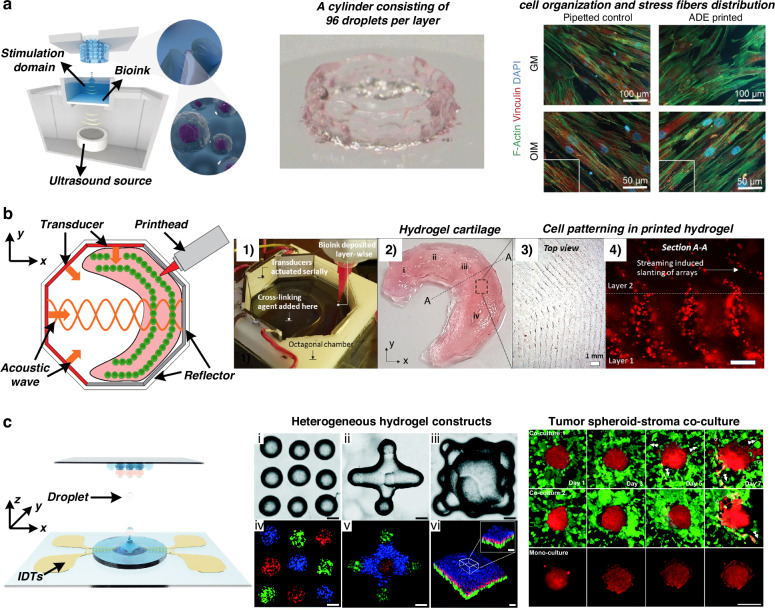
Acoustic-based bioprinting. **a** Acoustic droplet ejection minimizes critical shear stress in 3D bioprinting and demonstrates the creation of complex and individualized cell-laden 3D hydrogel structures. The figures were reprinted and modified from ref. ^103^ with permission from John Wiley and Sons. **b** Fabrication of a meniscus-like construct via acoustic 3D bioprinting. The figures were reprinted and modified from ref. ^105^ with permission from IOP Publishing. **c** Focused surface acoustic wave-based droplet printing for 3D biofabrication. Heterogeneous hydrogel constructs and tumor spheroid-stroma coculture models can be fabricated using different acoustic printers. The figures were reprinted and modified from ref. ^106^ with permission from the Royal Society of Chemistry

Recently, Jentsch et al.^103^ reported a novel 3D bioprinting method based on the principle of acoustic droplet ejection, as shown in Fig. 4a. The acoustic wave field is a virtual nozzle that avoids the physical geometry restrictions and critical shear stress induced by nozzles in traditional 3D printing. The authors demonstrated the construction of complex and individualized cell-laden 3D hydrogel structures with no adverse effects on stem cell morphology, proliferation, or differentiation capacities. Llewellyn-Jones et al.^104^ demonstrated the combination of acoustic technology and additive manufacturing. They printed cell-laden bioink three-dimensionally to control the shape and geometry of the structures and used acoustic manipulation to pattern and assemble the cells suspended in the bioink after printing but before polymerization. Chansoria et al.^105^ adopted this technology and developed an acoustic 3D bioprinting method, as shown in Fig. 4b. Chen et al.^106^ used focused acoustic waves to jet cell-laden droplets, as shown in Fig. 4c. They constructed a complex structure of a tumor spheroid surrounded by a high concentration of cancer-associated fibroblasts and found that acoustic 3D bioprinting resulted in greater cell viability than inkjet- and extrusion-based bioprinting methods. Later, the same group reported on the precise arrangement of colorectal cancer cells and healthy organoids using the acoustic 3D bioprinting method^107^. They constructed a tissue model mimicking the diseased colorectum of patients. Using this model, they conducted drug screening and assessed tumor invasion studies to improve cancer treatments and inform clinical decision-making. Acoustic 3D bioprinting wields the advantages of both bioprinting, which can formulate complex tissue macroarchitecture to mimic that of native tissues, and acoustic technology, which allows for a noncontact and label-free approach for the precise assembly of cells within the printed constructs.

## Acoustic-based in-scaffold control

Using scaffolds as supporting materials and templates has many advantages, including the feasibility of fabricating more complicated structures. After fabrication, cells are seeded into the scaffolds to culture, form an extracellular matrix, and eventually become an active tissue construct. Acoustic technologies are useful for engineering the properties of scaffolds during and after fabrication.

### Engineering fiber diameters via acoustics

**Fig. 5:**
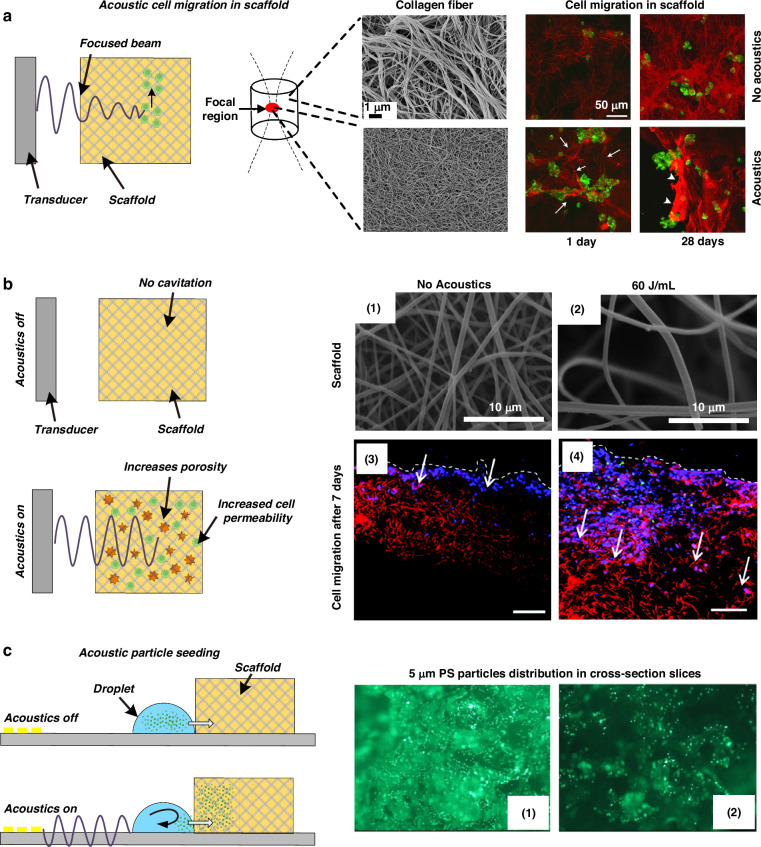
Acoustic technologies can be used to engineer scaffold properties. **a** An acoustic beam tunes the collagen fiber microstructure locally during scaffold fabrication, thus controlling cell migration behavior. The figures were reprinted and modified from ref. ^108^ with permission from the Acoustical Society of America. **b** Acoustic stimulation enhances the porosity of the scaffold postfabrication and thus controls cell migration behavior. The figures were reprinted and modified from ref. ^114^ with permission from Mary Ann Liebert, Inc. **c** Acoustic microstreaming induced by surface acoustic waves engineering particle migration mobility in a porous scaffold. Reprinted from ref. ^121^ with permission from Elsevier

In 2013, Garvin et al.^108^ reported the ability of acoustic waves to control collagen microstructure during hydrogel polymerization. They used a high-frequency (8.3 MHz) ultrasound beam to treat the hydrogel solution during polymerization and found that, under appropriate conditions, acoustic waves can decrease collagen fiber diameters compared to those of samples without acoustic treatment, as shown in Fig. 5a. The short, thin collagen fibrils induced by acoustic treatment make it easier for fibroblasts to migrate and form clusters. This work demonstrated that acoustic technologies can noninvasively and site-specifically control the microstructure of collagen fibrils and have the potential to produce scaffolds with defined mechanical and biological properties. Norris et al.^109,110^ thoroughly investigated the underlying mechanisms of this process and concluded that the heat produced by acoustic waves is not the key to modifying the collagen fiber structure. Mechanical forces associated with acoustic waves enhance both the functionality and bioactivity of collagen hydrogels. Collagen hydrogels, as well as skin explants obtained from diabetic mice, were subjected to acoustic exposure, and they all exhibited enhanced cell migration and cell-mediated collagen fiber remodeling^110^. In 2018, Nichols et al.^111^ demonstrated the use of acoustic waves to construct hydrogels with patterned coacervate microdroplets. The coacervate microdroplets were patterned in an acoustic field. After that, the hydrogel was added to the chamber to encapsulate the micropatterned coacervate droplets. Coacervate microdroplets present predominantly at the bottom led to spatially organized regions of dense hydrogelation and periodic patterns in the hydrogel monolith. This platform offers a method to synthesize hydrogels with unique and spatially patterned physical and chemical properties or spatially organized functional components.

### Engineering porosity of the scaffold via acoustics

Porosity is an essential parameter for effective tissue engineering scaffolds. Small pore size and low porosity may limit cellular infiltration, constrain mass transport, and hinder cell growth. Commonly, nanofibers or polymers in scaffolds are entangled and densely packed rather than shaped into porous 3D structures useful for tissue engineering. In 2006, Wang et al.^112^ used ultrasound to treat foamed biodegradable polymer scaffolds. The results demonstrated that ultrasound can substantially enhance interpore connectivity. In addition to the synthesized artificial scaffold, ultrasound treatment is also effective for natural tissue scaffolds^113^. In 2011, Lee et al.^114^ demonstrated that ultrasonic treatment greatly increased the pore sizes of electrospun nanofiber-based scaffolds, thereby enhancing cellular infiltration and proliferation, as shown in Fig. 5b. The pore size, porosity, and overall thickness of the nanofiber scaffolds can be adjusted by varying the exposure time and the energy of the ultrasound waves. Watson et al.^115^ reported that the structural porosity and fluidic properties of polymer foam tissue scaffolds were significantly enhanced upon exposure to acoustic waves. The water uptake increased from 40% to 100% after filling the scaffold, indicating more effective fluid transport and exchange. Gu et al.^116^ reported the increasing porosity of a chitosan mat via ultrasonic treatment. The treated chitosan scaffold promoted the proliferation of normal human dermal fibroblasts at a level that was 1.4-fold greater than that of the nontreated material after 7 days of culture. Similar effects were found for electrospun alginate nanofibers^117^ and CaCl_2_-polymerized alginate scaffolds^118,119^.

The underlying mechanism for porosity enhancement has been investigated. Lee et al.^114^ demonstrated that the increase in pore size is due to the mechanical separation of nanofibers via the vibrations of acoustic waves. Guo et al.^119,120^ proposed another opinion and designed experiments to obtain acoustic emission signals when the scaffolds were exposed to incident acoustic waves. They found sub- and ultraharmonic components with no obvious broadband noise spectrum. The results suggested that the violent acoustic cavitation and shock wave emitted from the collapse of bubbles might not be involved in the process. Based on this fact, they believed that shear stress arising from acoustic microstreaming was the primary mechanism for the enhanced porosity and permeability of the scaffolds.

Although the exact underlying mechanisms remain unclear and are currently under debate, acoustic waves show significant advantages in producing more porous scaffolds without damaging the overall architecture or significantly affecting the biochemical constituents.

### Accelerating particle seeding in scaffolds via acoustics

In addition to directly engineering scaffold properties using acoustics, Li et al.^121^ demonstrated the use of acoustics for quickly driving particles into a scaffold, providing a rapid, uniformly distributed, and efficient method that could be used to seed cells for tissue engineering applications. As shown in Fig. 5c, a porous scaffold is placed on the surface of an acoustic wave-based device next to a droplet containing particles. When surface acoustic waves are excited by the IDTs, the droplet is driven to penetrate the porous scaffold. This acoustic streaming-driven seeding process has been demonstrated to occur in approximately 10 s, which is significantly less than that of gravity-driven diffusional seeding processes alone (>30 min). Furthermore, an analysis of the particle distribution demonstrated that the acoustic-based method could also drive particles deeper into the scaffold.

## Opportunities for acoustic technologies to advance biofabrication and tissue engineering

With the progress reported in the past two decades, acoustic technologies have made significant advancements and yielded powerful toolsets for biofabrication and tissue engineering. Furthermore, it is notable that acoustic technologies, in combination with some emerging concepts and methods, have the potential to bring this field to new levels in the coming years. In this section, we speculate where the field will go in the next 10 to 20 years and introduce some of the most recent technological developments that can be applied to help resolve some of the current problems in biofabrication and tissue engineering.

### Acoustic mechanotransduction

Mechanical cues are comprehensively and profoundly associated with the whole tissue regeneration process^122^. Physical forces can regulate mechanosensitive systems, altering protein conformation to generate signals and further influencing the downstream pathways of mesenchymal stem cell differentiation^123,124^. In vitro models have demonstrated that a mechanical change induces different mesenchymal stem cell fates^125^. For example, a 2017 pilot study by Xue et al.^126^ used acoustic forces for mesenchymal stem cell mechanotransduction. They developed an acoustic tweezing cytometry method to actuate functionalized lipid microbubbles by ultrasound pulses. Lipid microbubbles exerted forces on mesenchymal stem cells and improved their osteogenic differentiation *via* integrin binding. With recent developments in acoustic technologies, direct control of force on a tiny area at the single-cell level is feasible. Acoustic forces have been used to pattern induced pluripotent stem cell cardiomyocytes^87^, but exploring how acoustic forces could affect the differentiation of induced pluripotent stem cells would be worthwhile. A comprehensive review of this topic is presented by Figarol et al.^127^.

Acoustic technologies can be used to assemble complex mesenchymal or induced pluripotent stem cell organoids and program them using acoustic signals for mechanical stimulation to drive their differentiation and generate personalized, viable tissue without ethical conflict.

### Manipulation of organoids via acoustics

Studying the communication and interactions among proteins widely present in biological systems, such as cells, tissues, and organs, is essential for many fields. Nevertheless, it needs to be improved due to the complexity of living objects and the need for an adequate toolset. In addition to constructing complex biological structures, acoustic technologies can dynamically manipulate constructed biotissues.

Ozcelik et al.^128^ and Meng et al.^129^ summarized various acoustic-tweezer technologies for dynamic control of objects. Briefly, acoustic tweezers have demonstrated the ability to manipulate objects ranging from several microns to several millimeters, e.g., extracellular vesicles, cells^26^, particles, droplets^130^, *C. elegans*^131^, and zebra fish^132^, with multiple orders of freedom (e.g., translational and rotational manipulation along the X, Y, and Z axes). Recently, Tian et al.^27^ developed a wavenumber–spiral surface acoustic tweezer platform to facilitate dynamic and programmable manipulation of cells and cell clusters. Baudoin et al.^133^ devised a holographic acoustic tweezer with high spatial selectivity in trapping objects to precisely move them in any direction in the plane. Ghanem et al.^134^ demonstrated complex 3D in vivo manipulation of glass spheres using a single acoustic transducer, alluding to the potential of acoustic tweezers for applications in vivo. Chen et al.^135^ demonstrated the controllable rotation of organisms (e.g., zebrafish via acoustic streaming). Zhu et al.^136^ recently developed a digital acoustofluidics platform for programmable manipulation of cells and droplets. Yang et al.^137^ developed a flexible manipulation platform for living cells and organisms. The platform utilized a 64-element planar ultrasound transducer array to generate a focusing vortex and twin fields based on the holographic acoustic element framework method, thus enabling multidimensional translation, rotation, orientation, and levitation. Gao et al.^138^ reported the assembly of organoids using an ultrasonic 2D matrix phase array. They developed a platform with precise selection, movement, rotation, and accurate combination of organoids and demonstrated the construction of heterogeneous assembloids.

Therefore, the use of acoustic technologies to control organoid movements and organoid–organoid interactions is a promising research direction. Acoustic technologies can dynamically control vesicles, from cells to large-scale tissue or organoids, across a wide range of sizes, from nanometers to millimeters. Additionally, they can manipulate the motion of objects in three dimensions with six degrees of freedom, encompassing translation, rotation, and mixed movements. Additionally, the contactless nature of the acoustic waves helps maintain the integrity of the biospecimen during the procedure. The acoustic-based devices are highly versatile and can manipulate objects in various environments, including the native fluids of living objects. Compared to other contactless manipulation counterparts, such as optical tweezers, electrophoresis, and magnetic tweezers, acoustic tweezers are more biocompatible, concise, and powerful for manipulating biological objects^139–146^.

### Subtractive fabrication via acoustics

Tissue ablation is another potential application of acoustic technologies in biofabrication and tissue engineering. The high-resolution acoustic ablation technique is a subtractive fabrication method that could be useful for removing unwanted parts of organoids, such as peeling off the outer layer of an organoid or some part of a tissue for quality control or real-time monitoring, or facilitating the construction of complex multicellular architectures. In addition, a more critical application is the fabrication of a complex vasculature network in organoids. A significant limitation for in vitro models in achieving truly in vivo-like functionality is the need for organoid vascularization^147^. A complex vasculature network allows the exchange of oxygen, nutrients, and waste and provides a structural template for growth. However, methods for forming vasculature inside organoids still need to be improved, and many disadvantages exist^147^. Methods such as bioprinting and sacrificial networks are limited because the constructed structures cannot be modified after their initial creation. Laser-based ablation allows flexibility after organoid formation. However, laser ablation is limited to the horizontal plane because it removes the path of the laser within the whole organoid. Acoustic ablation could resolve these limitations by leveraging the ability to create genuinely biomimetic, vascularized organoids.

Currently, acoustic-based ablation techniques are widely employed clinically in vivo to remove lesions and treat cancer and gynecological diseases^23,148^. High-intensity focused ultrasound (HIFU) is generally used in acoustic-based methods to generate transient thermal effects and ablate tissues. The frequency range of ultrasound used during clinical applications is typically 0.8–3.5 MHz, and the volume of ablation, which is the focal area of HIFU, can vary according to the ultrasound frequency and the transducer dimensions but is typically on the order of 1–3 mm (transverse) × 8–15 mm (along the beam axis) to match the tumor volume^149^. In contrast, in vitro applications such as biofabrication and tissue engineering can use higher-frequency ultrasound and microfabricated transducers to achieve much finer resolution. Therefore, with adequate modifications, acoustic technologies can potentially become tools for generating organoids.

## Summary

In the past 20 years, the number of studies using acoustic technologies for biofabrication and tissue engineering has rapidly increased. In this review, we introduced the basic mechanisms of acoustic-based technologies and reviewed the developments and applications of acoustic technologies in various aspects of biofabrication and tissue engineering. We focused on acoustic-based cell assembly and acoustic-assisted scaffold fabrication. For cell assembly, we tailored the practice of various acoustic technologies to fabricate biostructures summarized in four categories: cell spheroid clusters, vessel-like constructs, membrane-like constructs, and complex geometry constructs. A comparison of these acoustic technologies in terms of the precision of control, tunability of structure, and throughput of fabrication is listed in Table 3. We also discussed follow-up applications in tissue engineering. We reviewed the progress in engineering scaffold properties using acoustic technologies for fabrication. Finally, we provided a perspective based on the most recent developments in acoustics and speculated on possible future directions for this field, hoping to inspire the research community.

**Table 3:** Comparison of different acoustic biofabrication technologies

	*Precision of control*	*Tunability of structure*	*Throughput of fabrication*
Bulk acoustic wave	1–2 cells wide^84^	Spheroid-like, vessel-like, membrane-like	~13,000 spheroids per operation^64^
Surface acoustic wave	Single-cell level^25^	Spheroid-like, vessel-like, membrane-like	~6000 spheroids per operation^62^
Acoustic microstreaming	30 μm^68^	Spheroid-like	<100 spheroids per operation^67^
Faraday wave	>100 μm^86^	Complex structure	96 constructs per operation^88^
Acoustic 3D bioprinting	~20 μm^106^	Arbitrary shape	~1000 spheroids per operation, ~1 h for printing a 10 × 10 × 10 mm^3^ construct^106^
Acoustic holography	Single-cell level^95^	Arbitrary shape	2–3 min to form a pattern^94^

Acoustic technology has significantly advanced the fabrication of multicellular biostructures, utilizing individual cells and biomaterials such as hydrogels for support. Constructs such as multicellular spheroids, vessels, and complex arbitrary structures have been successfully demonstrated. However, each type of structure requires different experimental setups, and tailored engineering design, such as the arrangement and geometry of acoustic transducers, requires advanced expertise and fabrication skills. Thus, fabrication flexibility remains a challenge for acoustic technology. As a comparison, 3D bioprinting methods can construct tissues and organs with complex geometries and structures on demand via computer programming. Significant efforts are still needed to make acoustic technology more accessible to the biofabrication and tissue engineering community.
